# A 28-Day, Randomized, Controlled, Single-Blind, Phase 2 Study in Treatment-Resistant Major Depressive Disorder (TRD) Patients Receiving Intranasal Esketamine Comparing Addition of Almond Therapy TM with Treatment-as-Usual (TAU)

**DOI:** 10.1192/j.eurpsy.2023.903

**Published:** 2023-07-19

**Authors:** P. Chue, P. Silverstone, T. Hillier, S. Rizvi, S. Phillips, L.-A. Langkaas, K. Davidson, M. Brown, J. Chue

**Affiliations:** 1 University of Alberta; 2 Zylorion; 3 Amygdala Associates, Edmonton, Canada

## Abstract

**Introduction:**

Treatment Resistant Depression (TRD) occurs in up to 30% of patients with Major Depressive Disorder (MDD). New treatments are clearly needed and there is a burgeoning interest in novel agents including ketamine. While ketamine in various formulations has been demonstrated to have a robust antidepressant effect there is a lack of evidence-based psychotherapies specifically designed for combination use.

**Objectives:**

We hypothesize that the combination of “Almond TherapyTM” with intranasal ketamine will result in a statistically significantly better outcome as demonstrated by a greater reduction in MADRS scores and/or response rates and/or remission rates in TRD patients compared with those who receive esketamine plus TAU. Secondary outcome measures include PHQ-9, GAD-7, PCL-5, Asssessment of Quality of Life - 8D (AQOL-8D), and Rosenberg Self-Esteem Scale.

**Methods:**

We have developed a research protocol combining a unique and specifically-designed, multi-modal psychotherapy program, “Almond TherapyTM”, with intranasal esketamine in a randomized, controlled, single-blind 28-day study. The therapy utilizes an individualized, evidence-informed approach for each participant consisting of a number of modules selected using a shared decision-making process determined at the first study visit. This uniquely tailored approach ensures that the chosen modules are personally meaningful to the participant, and thus, promotes therapeutic adherence. The proprietary therapy combines elements of cognitive behavioral therapy (CBT), trauma focused-CBT, Dialectical Behavioral Therapy (DBT), and mindfulness, together with biofeedback. In addition to in-clinic sessions, participants also receive standardized remote therapy sessions by trained therapists.

**Results:**

Patient recruitment and enrolment has begun. Interim results are anticipated.

**Conclusions:**

This study is the first examination of the potential additional clinical benefit of adding a specific therapy program to existing intranasal esketamine treatment. If demonstrated to be of clinical benefit then further studies may potentially provide comparison to other therapy programs and in conjunction with other agents.

**Disclosure of Interest:**

P. Chue Shareolder of: Zylorion, P. Silverstone Shareolder of: Zylorion, Employee of: Zylorion, T. Hillier Shareolder of: Zylorion, Employee of: Zylorion, S. Rizvi: None Declared, S. Phillips Shareolder of: Zylorion, Employee of: Zylorion, L.-A. Langkaas Employee of: Zylorion, K. Davidson Employee of: Zylorion, M. Brown: None Declared, J. Chue: None Declared

